# Experimental Investigation on the Creep Property of Carbon Fiber Reinforced Polymer Tendons under High Stress Levels

**DOI:** 10.3390/ma11112273

**Published:** 2018-11-14

**Authors:** Dong Yang, Jiwen Zhang, Shoutan Song, Fei Zhou, Chao Wang

**Affiliations:** 1 School of Civil Engineering, Southeast University, Nanjing 210096, China; dongyang@seu.edu.cn (D.Y.); songshoutan@seu.edu.cn (S.S.); zhoufei0912@seu.edu.cn (F.Z.); chao.wang.seu@gmail.com (C.W.); 2 Key Laboratory of Concrete and Prestressed Concrete Structures of the Ministry of Education, Southeast University, Nanjing 210096, China

**Keywords:** carbon fiber reinforced polymer (CFRP), long-term property, creep

## Abstract

Carbon fiber reinforced polymer (CFRP) tendons are generally used as prestressing members to take full advantage of their high strength. Their creep property is one of the key factors influencing the reliability and safety of the structures, especially under sustained high stress. In this study, using a new wedge-type anchorage system, experimental research was carried out on the creep behavior of CFRP tendons under high stress levels from 0.69 to 0.85 *f_u_*. All the tests lasted for a duration of 1000 h. It was found that the creep coefficient tends to increase with the stress level. Compared to their static properties, the residual strength of CFRP tendons after creep tests is 4.54% lower while the after-creep elastic modulus is 6.99% higher. Through data analysis, a semi-logarithm linear relationship between the creep coefficient and time was established, and the creep coefficients at 1 million hours were extrapolated.

## 1. Introduction

Fiber reinforced polymer (FRP) materials are characterized by many excellent properties, such as low weight, high strength, corrosion resistance, and non-magnetism. They have shown promising application potential for building retrofitted and concrete structures under a corrosive environment [1,2,3]. At present, the most commonly used FRP materials are aramid FRP (AFRP), glass FRP (GFRP), basalt FRP (BFRP), and carbon FRP (CFRP). Among all the FRP materials, CFRP exhibits the best performance and is the most ideal substitute material for steel.

Owing to their high strength and relatively low elastic modulus, CFRP tendons are a common preference in civil engineering for use as prestressing members to take full advantage of their high performance [4]. However, the creep property of CFRP materials may lead to the stress redistribution of different structural components, subsequently influencing the reliability and durability of the structures [5,6]. For civil engineering structures, including bridges, the designed service life is generally over 50 years. The lack of understanding of the creep property of CFRP materials has partly limited the application of these materials.

Besides this, due to the low transverse strength and delicate surface of CFRP tendons, a feasible and reliable anchorage system for the CFRP tendons under sustained load is also one of the key problems to be solved. A new wedge-type anchorage system was developed by the authors’ research group and was used in this study for a series of tests on the creep behavior of CFRP tendons. The long-term performance of the anchorage system was also investigated.

## 2. Literature Review

Creep is the outward manifestation of FRP materials’ viscoelastic property. It is induced by three circumstances: (a) the straightening of uneven fibers, (b) the viscoelastic deformation of the resin matrix, and (c) the creep of fibers [7]. A lot of studies have already been carried out on CFRP tendons [8,9]. However, only limited references are available on the long-term behavior of CFRP tendons in the current literature. The previous research on the creep behavior of CFRP tendons can be divided into two categories: research on the creep rupture properties and research on the creep strain under different stress levels. Yamaguchi [10] studied the creep rupture phenomenon of CFRP rods and revealed a semi-logarithm relationship between the creep rupture stress and time. Through extrapolation, he determined the creep rupture strength of CFRP rods after 1 million hours to be 0.92 fu. Ando [11] found the creep rupture behavior of twisted CFRP bars to be independent of the sectional dimension and calculated the creep rupture strength to be 0.79 fu after 50 years. Hamid Saadatmanesh [12] tested the creep performance of both CFRP tendons (Leadline) and twisted CFRP cables (CFCC) subjected to stress levels of 0.4 fu. No creep was observed for Leadline, and the creep strain of the CFCCs at 3000 h was 0.015%. Patrick X. W. Zou [13] also reported the creep coefficient of CFRP tendons (Leadline) to be zero for stress levels below 0.6 *f_u_*. Xiong Yang [14] conducted a series of creep tests on CFRP cables at 95% of their guaranteed capacity for 5 months. Neither rupture nor any sign of distress was observed. The residual strength of the specimens was 14% lower than the static strength but still higher than the guaranteed capacity.

It can be concluded from the literature cited above that CFRP tendons possess a high creep rupture strength and low creep coefficient. However, a relatively large discrepancy exists in the creep property of CFRP tendons provided by different manufactories. Even when using identical tendons, the conclusions drawn by different researchers are not exactly consistent. In addition, little data on the creep coefficient of CFRP tendons are available, especially for high stress levels over 0.60 *f_u_*.

On the other hand, there is also continuing concern regarding efficient and reliable anchorages for CFRP tendons. There are two main types of anchorages for CFRP tendons at present: the bonded anchorage and mechanical anchorage. Bonded anchorages come with the disadvantages of long curing times, high slippage, and long lengths. In contrast, the mechanical anchorages are preferred for structural applications because they are easy to mount [15]. Several mechanical anchorages have been developed for CFRP tendons, such as the split-wedge anchorage, nonmetallic wedge anchorage, and integrated sleeve-wedge anchorage [16]. In 2002, split-wedge anchorages were applied to the Laroin CFRP Footbridge for anchoring CFRP cables [17]. However, as a compressive force perpendicular to the tendon exists in mechanical anchorages, premature failure or local crushing at the anchorage zone is likely to take place [15]. Mechanical anchorages possessing reliability and stability still remain to be developed.

Based on the new wedge-type anchorage system developed by the authors’ research group, in this study, a series of creep tests were carried out under stress levels higher than 0.60 *f_u_* with a duration of 1000 h. The creep coefficient, residual strength, and elastic modulus after creep were measured. The creep coefficients of CFRP tendons at 50 years and 1 million hours were predicted from 1000 h of test data.

## 3. Materials and Methods

### 3.1. Specimen Preparation and Anchoring System

All the tests were performed at the Testing Laboratory of Civil Engineering in Southeast University. The CFRP tendons used in this study are commercially available and were provided by Jiangsu Hengshen Co. Ltd. (Zhenjiang, China). These tendons, with a nominal diameter of 8 mm and a smooth surface, were manufactured by pultrusion technology using carbon fibers and epoxy resin. The fiber volume fraction of the CFRP tendons was approximately 65%.

**Figure 1:**
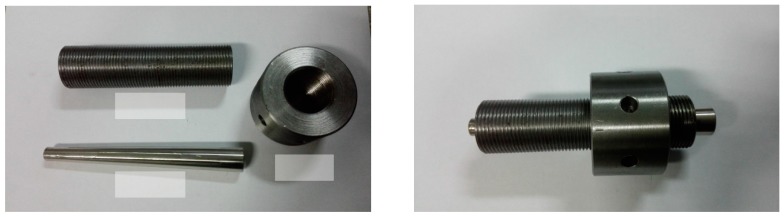
Wedge-type anchorage for the tests.

Figure 1 presents the new wedge-type anchorage system, which was dedicated to the CFRP tendons used in the tests. The anchorage consists of a wedge, a barrel, and a nut; the soft sleeve used in common wedge-type anchorages is omitted [15,18,19]. Specifically, a wedge with four notches is integrated and its inner face is smooth. It is able to grip the tendon directly without injuring the material surface. Static and fatigue tests were conducted in previous research, and this type of anchorage showed a reliable performance [20].

### 3.2. Testing Setups

#### 3.2.1. Static Test Setup

**Figure 2:**
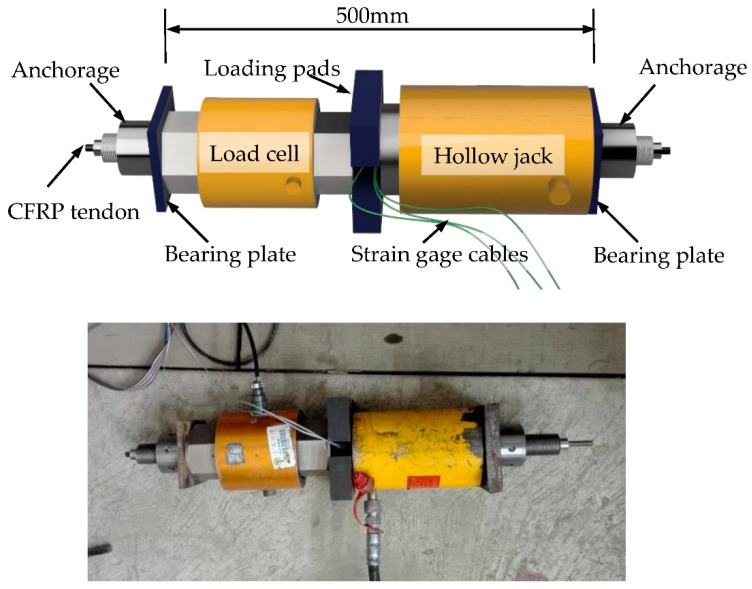
Static test setup.

The static tensile strength test setup consists of a hollow jack, a load cell, bearing plates, and loading pads, as shown in Figure 2. Three strain gauges were attached to the middle of each tendon to measure its strain. The original load and strain data were captured by a data acquisition instrument. All the specimens were loaded to failure, and the data of five specimens which did not fail at the anchorage zone were picked out to determine the static tensile strength.

#### 3.2.2. Creep Test Setup

**Figure 3:**
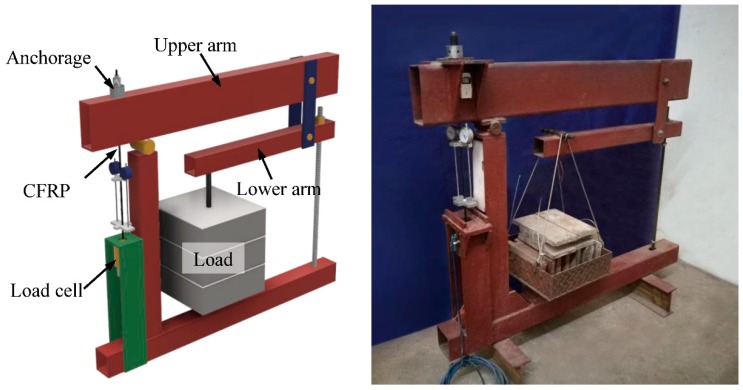
Creep test frame.

To maintain the load stability during the creep test on CFRP tendons, a test frame was designed and fabricated for this study (Figure 3). The frame was able to magnify the applied load by a factor of 56 through the application of the lever principle. A screw was installed at the end of the lower arm to adjust the alignment of two horizontal arms. The precise load applied to the CFRP tendon was measured by a load cell that was set at the anchorage zone.

**Figure 4:**
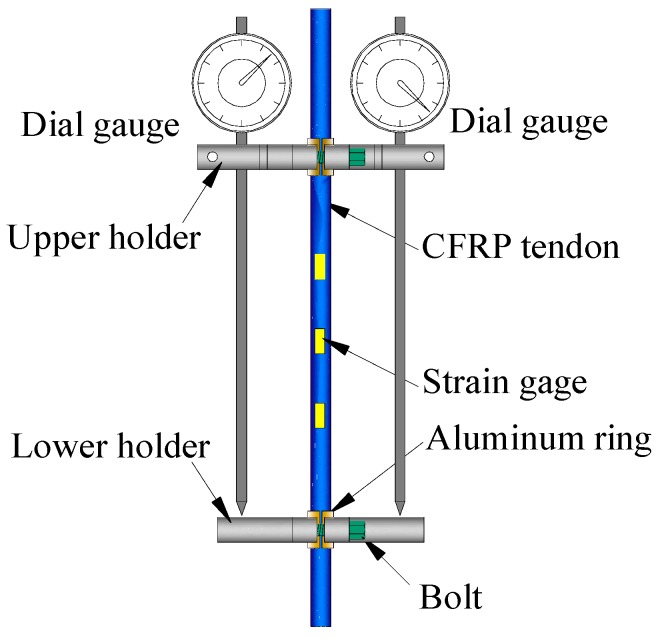
Strain measurement device.

To measure the strain of the specimen over a long observation period, a dedicated device was used as shown in Figure 4. This device contains an upper holder, a lower holder, and two dial gauges. Both the upper holder and lower holder were divided into two symmetric parts which were connected and fixed to the tendon by bolts. As the CFRP tendon has a low transverse strength, two aluminum rings were installed between holder parts to wrap the tendon to prevent the specimen from being damaged by the extrusion force. The distance between the upper holder and lower holder was 220 mm and the dial gauges had a precision of 0.001 mm. So, the precision of this measurement device was calculated to be A = 0.001/220 = 44.5 × 10^−6^ = 4.5 με. To verify the reliability of this device, three strain gauges were attached to the middle of the first specimen. The data obtained from both the device and the strain gauges were recorded during the loading process. The comparison showed that the deviation between these two data sets was less than 5%, demonstrating the efficiency of this strain measurement device.

### 3.3. Loading Procedure

#### 3.3.1. Static Test Loading Procedure

For static tensile strength tests, the loading rate was set to be 200 MPa/min. Both the load and strain data were recorded by the data acquisition instrument every second until the failure of the specimen. Five effective specimens without failure at the anchorage zone were used to determine the static tensile strength of the CFRP tendons which served as a reference in the later creep tests. The static elastic modulus was calculated from the data points from 20 and 50% of the ultimate tensile strength.

#### 3.3.2. Creep Test Loading Procedure

The long-term behavior of CFRP tendons has been proved to be influenced by external conditions, such as alkaline and acidic environments and temperature [12]. Limited by experimental conditions, in this work, the creep tests were carried out only at room temperature in air. Three groups of creep tests were conducted, and each group consisted of three specimens. The specimen ID and corresponding stress levels are listed in Table 1. The mass blocks were added block by block slowly so as to ensure that the increase rate of the stress in the tendon was approximately 200 MPa/min [21]. Once the prescribed stress value was attained, the data were recorded at the following times: 1, 3, 6, 9, 15, 30, 45 min and 1, 1.5, 2, 4, 10, 24, 48, 72, 96, 120 h. Subsequent measurements were taken once every 120 h. After 1000 h of the creep test, the specimens were taken down for tensile strength tests to measure the residual strength and elastic modulus.

## 4. Results and Discussion

### 4.1. Static Tensile Properties

**Figure 5:**
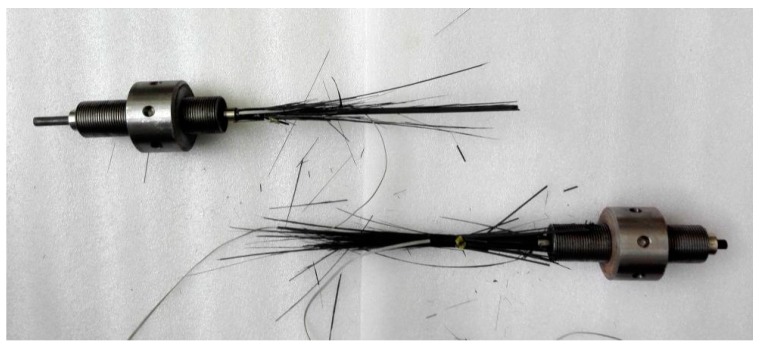
Failure mode of CFRP tendons.

All the specimens for static tensile tests failed in a similar mode, as shown in Figure 5. Instantaneous fracture at the middle region took place and was accompanied by a loud sound, and the complete dispersal of CFRP fibers could be observed. No premature failure or slippage at the anchorage zone was observed, which verified the efficiency of this anchorage system. The static tensile test results are listed in Table 2. The mean values of the tensile strength and elastic modulus are 2159 MPa, with a variation coefficient of 4.1%, and 147.3 GPa, with a variation coefficient of 0.95%. The small variation coefficients demonstrate the stability of the CFRP tendons. For design considerations, a 95% guaranteed strength of 2012 MPa is derived from Equation (1) [22].

(1) $$f_{uk}=\bar{f_{u}}-1.645\sigma_{f}$$

where $f_{uk}$, $\bar{f_{u}}$ and $\sigma_{f}$ are the 95% guaranteed strength, mean value, and standard deviation of the ultimate strength, respectively.

### 4.2. Creep Properties

#### 4.2.1. Creep Curve

**Figure 6:**
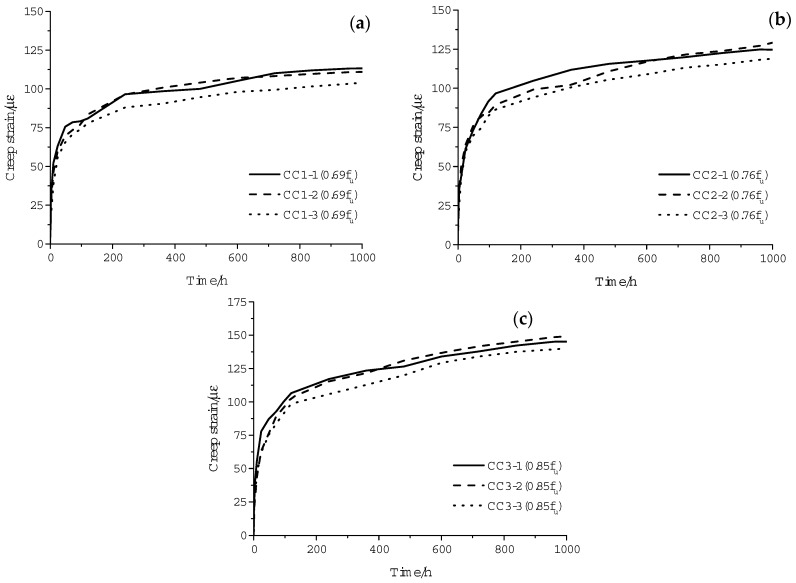
Strain–time curve at stress levels of (**a**) 0.69 *f_u_*, (**b**) 0.76 *f_u_* and (**c**) 0.85 *f_u_*.

The strain of each specimen recorded over time is plotted in Figure 6. A continuous increase in strain is characterized for all specimens. Since no creep rupture phenomenon was observed in any of the creep tests, the *ε*–*T* relationships are characterized by two stages instead of the typical three stages [11]. At the first stage, the strain increases at a relatively rapid speed. Then, the rates of creep slow down gradually and go into the second stage, which is stable and lasts for a long time. This development law is in accordance with the results observed in creep tests of other composite materials [23]. Furthermore, the specimens under higher creep stress levels are characterized by larger creep strains. This is because higher stress leads to straighter fibers and larger viscous deformation of the resin matrix. Several fluctuations can be observed in the creep curves, which are caused by the measurement error and thermal deformation of the testing device.

#### 4.2.2. Creep Coefficient

The creep coefficients, defined as the ratio of creep strain to elastic strain, of the specimens under different stress levels at 1000 h are listed in Table 3. A comparison of the results reveals the tendency of a slightly higher creep coefficient under a higher load level. The variation coefficients of all three test groups are lower than 5%, and its value decreases with as the load level increases. This observation is similar to that in the creep tests of BFRP (basalt fiber reinforced polymer) tendons under high stress levels [23]. A defect expansion mechanism was developed and gives a reasonable explanation for this phenomenon. With the increase in stress, the development of the original defects in the FRP material becomes more sufficient, and this appears as better uniformity of the specimens [23]. The test results in this paper can also be explained by this theory.

#### 4.2.3. Residual Tensile Strength and after-Creep Elastic Modulus

To assess the influence of sustained stress on the mechanical properties of CFRP tendons, all the creep specimens were reloaded to failure after the creep tests. The failure modes are the same as those in static tensile tests. No premature failure at the anchorage zone was observed, which illustrates that the performance of the anchorage did not degrade under a sustained load. From the test results listed in Table 4, we can see that compared with the static tensile test results, the average residual tensile strength (2061 MPa) decreases by 4.54%, while the average after-creep elastic modulus (157.6 GPa) increases by 6.99% and the rupture strain (1.28%) decreases by 12.9%. Moreover, no significant trend can be found for the residual properties of the specimens treated at different stress levels. This phenomenon can be explained as follows. Sustained high stress is likely to cause damage to the interface between the fibers and resin, and the damage would expand rapidly under high stress near failure and weaken the collaboration between the fiber and resin matrix, leading to a decrease in residual strength [24]. Meanwhile, the original uneven fibers were straightened during the creep tests; thus, the collaboration of the fibers improves and the after-creep elastic modulus increases. As for the specimens CC1–CC3, based on previous creep rupture research on CFRP tendons [10,11], no creep rupture is likely to take place in a short period of time under the designed creep stress levels. This means that the damage evolution inside the material in previous creep tests was stable, and the deterioration of all the specimens was restrained to a certain degree, resulting in a slight discrepancy in the residual properties.

#### 4.2.4. Creep Coefficient Prediction

**Figure 7:**
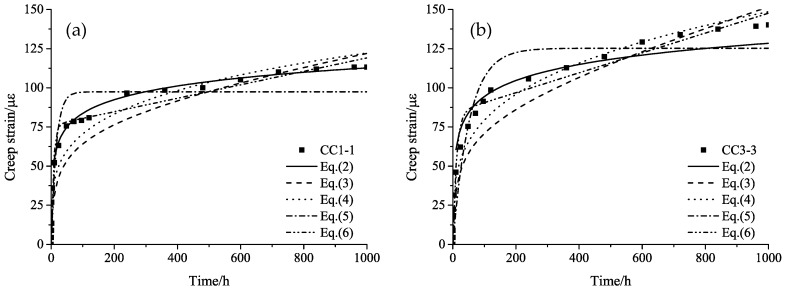
The fitting curves of different models for specimens (**a**) CC1-1 and (**b**) CC3-3.

Although various models have been developed for the creep behavior of plastic materials [25], a universally applicable model for the prediction of creep behavior in FRP materials is still lacking. Equations (2)–(6) are several classical creep relations [13,25] and are compared to the creep test results. Due to limited space, only the comparison results for specimens CC1-1 and CC3-3 are plotted in Figure 7.

(2) $$\varepsilon_{c}=\varepsilon -\varepsilon_{0}=a\ln t+b$$

(3) $$\varepsilon_{c}=\varepsilon -\varepsilon_{0}=a\ln t+bt$$

(4) $$\varepsilon_{c}=\varepsilon -\varepsilon_{0}=at^{b}$$

(5) $$\varepsilon_{c}=\varepsilon -\varepsilon_{0}=a(1-e^{-bt})$$

(6) $$\varepsilon_{c}=\varepsilon -\varepsilon_{0}=a(1-e^{-bt})+ct$$

where *t* is the time in hours, *ε_c_* and *ε* are the creep strain and total strain at time *t*, respectively, *ε*_0_ is the initial elastic strain, and *a*, *b*, and *c* are empirical constants.

It can be seen from Figure 7a that the fitting curves of Equation (2) are very close to the test data. The fitting curves of Equations (3) and (4) are similar and underestimate the creep strain at the earlier stage, while they overestimate it latterly. A rapid increase in creep strain is observed at the beginning of the curve of Equation (5), and the strain becomes stable after 100 h, which does not match the test data. The curve of Equation (6) fits well with the test data at the first stage, but it becomes almost linear afterward. The calculated strain will be far larger than the actual value at the later stage.

The fitting result in Figure 7b is similar to that in Figure 7a, in general. However, the curve of Equation (2) obviously underestimates the strain at the later stage.

The fitting results for specimens CC1-2, CC1-3, CC2-1, CC2-2, CC2-3, and CC3-1 are similar to that for CC1-1, and the result for specimen CC3-2 is similar to that of CC3-3. From a comparison, we can see that for specimens CC3-2 and CC3-3, the increase rate of the creep strain at the second stage are obviously higher than those of other specimens. From the creep rupture test results in previous research in References [10,11], we can speculate that the stress level of 0.85 *f_u_* may have been close to or already exceeding the creep rupture stress of the CFRP tendons. Under such a sustained high stress level, parts of the fibers start to fracture gradually, resulting in the stress and strain increase in the maintained fibers. Given a longer test duration, creep rupture might take place in these tests.

Based on the above analysis, Equation (2) fits the creep strain test data with the best precision. This calculation model considers the creep coefficient of CFRP tendons to be linear with the logarithm of time, which is reasonable and agrees with the research conclusion drawn by Patrick X. W. Zou [13] from the creep study of AFRP tendons. It can be rewritten as Equation (7) for the long-term creep coefficient prediction of CFRP tendons.

(7) $$\varphi (t)=\varepsilon_{c}/\varepsilon_{0}=a\ln t/\varepsilon_{0}+b/\varepsilon_{0}$$

Constants *a* and *b* for a certain load level can be determined by regression analysis of the corresponding data obtained during the 1000 h creep test. Then, the creep coefficients of the CFRP after 1000 h can be predicted by substituting *a* and *b* into Equation (7). The prediction results are listed in Table 5.

As can be seen from Table 5, the difference between the predicted creep coefficients of the CFRP tendons at 50 years and 1 million hours under sustained stresses from 0.69 *f_u_* to 0.85 *f_u_* is small. Basically, this phenomenon stays the same as that observed in the results of the 1000 h test. According to the analysis in Figure 7, it can be speculated that the predicted creep values for stress levels of 0.69 *f_u_* and 0.76 *f_u_* are reliable, while a considerable underestimation can be foreseen for the creep prediction for stress level of 0.85 *f_u_*. Still, more tests with longer duration times are necessary for the verification of this conclusion.

## 5. Conclusions

In this study, an experimental study was carried out on the creep property of CFRP tendons with a wedge-type anchorage that was developed by the authors’ research group. The main conclusions are as follows.

(1) The creep curves for all the specimens under sustained stresses from 0.69 *f_u_* to 0.85 *f_u_* are similar and can be divided into two stages. The creep strain increases quickly in the first stage and becomes stable in the second stage. Generally, a higher creep stress leads to a larger creep coefficient. The creep coefficients at 1000 h vary from 1.08 to 1.16%.
(2) Compared to their static properties, the CFRP tendons’ residual strength after creep tests of 1000 h is 4.54% lower, while the after-creep elastic modulus is 6.99% higher.
(3) A semi-logarithm relationship is found to fit the creep data within 1000 h with an acceptable precision. Through the extrapolation of this relationship, the creep coefficients at 1 million hours under stress levels of 0.69–0.85 *f_u_* are 1.83–1.91%. However, under stress levels close to or exceeding the creep rupture stress of CFRP tendons, the creep coefficients predicted by this relationship are smaller than the actual values.
(4) No premature failure at the anchorage zone was observed in any of the tests. The 8 mm wedge-type anchorage developed by the authors’ research group performed well in both the static and creep tests.

## Figures and Tables

**Table 1:** Load levels for the creep tests.

Creep Specimen ID	Creep Stress Level
CC1-i (i = 1~3)	0.69 *f_u_*
CC2-i (i = 1~3)	0.76 *f_u_*
CC3-i (i = 1~3)	0.85 *f_u_*

Note: In column 1 (creep specimen ID), the first letter “C” represents carbon fiber reinforced polymer (CFRP) and the second letter “C” represents creep tests. “*f_u_*” represents the ultimate tensile strength of the CFRP tendons.

**Table 2:** Static test results.

	Tensile Capacity/kN	Tensile Strength/MPa	Elastic Modulus/GPa	Rupture Strain/%
Mean value	108.6	2159	147.3	1.47
Standard deviation	4.51	89.57	1.39	0.054
Variation coefficient	0.041	0.041	0.0095	0.037

Note: In column 1 (specimen ID), the first letter “C” represents CFRP and the second letter “S” represents static tests.

**Table 3:** Creep coefficient (CC) of CFRP specimens.

Specimen ID	CC1 (0.69 *f_u_*)			CC2 (0.76 *f_u_*)			CC3 (0.85 *f_u_*)		
	1	2	3	1	2	3	1	2	3
Creep coefficient (1000 h, %)	1.12	1.10	1.03	1.11	1.15	1.06	1.19	1.16	1.13
Mean value (%)	1.08			1.11			1.16		
Standard deviation (%)	0.047			0.045			0.030		
Variation coefficient	0.0436			0.0407			0.0259		

**Table 4:** Residual tensile strength and after-creep elastic modulus.

	Tensile Capacity/kN	Tensile Strength/MPa	Elastic Modulus/GPa	Rupture Strain/%
Mean value	103.7	2061	157.6	1.28
Standard deviation	4	79.5	1.88	0.047
Variation coefficient	0.039	0.039	0.012	0.037

**Table 5:** Constant values and predicted creep coefficient.

Specimen ID		Constants		Regression Coefficient	Creep Coefficient (%)			
		*a*	*b*		50 Year	Mean Value	10^6^ h	Mean Value
CC1 (0.69 *f_u_*)	1	12.14	25.50	0.966	1.81	1.75	1.91	1.84
	2	12.07	24.67	0.965	1.79		1.89	
	3	11.09	22.94	0.959	1.65		1.74	
CC2 (0.76 *f_u_*)	1	13.04	29.70	0.967	1.77	1.74	1.87	1.83
	2	12.99	28.56	0.962	1.76		1.85	
	3	12.46	24.94	0.958	1.66		1.76	
CC3 (0.85 *f_u_*)	1	15.32	31.11	0.964	1.84	1.81	1.94	1.91
	2	15.85	26.54	0.943	1.86		1.96	
	3	14.76	26.44	0.952	1.74		1.84	

## Supplementary Materials

The following are available online at http://www.mdpi.com/1996-1944/11/11/2273/s1.
